# Computer-aided diagnosis system versus conventional reading system in low-dose (< 2 mSv) computed tomography: comparative study for patients at risk of lung cancer

**DOI:** 10.1590/1516-3180.2022.0130.R1.29042022

**Published:** 2022-10-28

**Authors:** Dong Wang, Lina Cao, Boya Li

**Affiliations:** I MD. Physician, Department of Medical Imaging, Xianyang Cai-Hong Hospital, Xianyang, Shaanxi, China.; II MD. Physician, Department of Medical Imaging, Hospital of Shaanxi University of Chinese Medicine, Xianyang, Shaanxi, China.; III MD. Physician, Department of Medical Imaging, Jiangxi provincial People’s Hospital, The First Affiliated Hospital of Nanchang Medical College, Nanchang, Jiangxi, China.

**Keywords:** Diagnostic imaging, Early detection of cancer, Lung neoplasms, Cancer nodule, Computed tomography, Computer-aided detection system, Image plane, Lung cancer, Radiation dose

## Abstract

**BACKGROUND:**

Computer-aided diagnosis in low-dose (≤ 3 mSv) computed tomography (CT) is a potential screening tool for lung nodules, with quality interpretation and less inter-observer variability among readers. Therefore, we aimed to determine the screening potential of CT using a radiation dose that does not exceed 2 mSv.

**OBJECTIVE:**

We aimed to compare the diagnostic parameters of low-dose (< 2 mSv) CT interpretation results using a computer-aided diagnosis system for lung cancer screening with those of a conventional reading system used by radiologists.

**DESIGN AND SETTING:**

We conducted a comparative study of chest CT images for lung cancer screening at three private institutions.

**METHODS:**

A database of low-dose (< 2 mSv) chest CT images of patients at risk of lung cancer was viewed with the conventional reading system (301 patients and 226 nodules) or computer-aided diagnosis system without any subsequent radiologist review (944 patients and 1,048 nodules).

**RESULTS:**

The numbers of detected and solid nodules per patient (both P < 0.0001) were higher using the computer-aided diagnosis system than those using the conventional reading system. The nodule size was reported as the maximum size in any plane in the computer-aided diagnosis system. Higher numbers of patients (102 [11%] versus 20 [7%], P = 0.0345) and nodules (154 [15%] versus 17 [8%], P = 0.0035) were diagnosed with cancer using the computer-aided diagnosis system.

**CONCLUSIONS:**

The computer-aided diagnosis system facilitates the diagnosis of cancerous nodules, especially solid nodules, in low-dose (< 2 mSv) CT among patients at risk for lung cancer.

## INTRODUCTION

Low-dose computed tomography is an effective imaging modality to reduce mortality in patients at high risk of lung cancer.^1,2,3,4^ In China, the computed tomography interpretation systems for the management of lung cancer vary among institutions. Moreover, the experiences of radiologists have an impact on computed tomography interpretation.^5^ Therefore, standardized computed tomography interpretation and management of nodule screening is crucial.^6,7,8,9^

Computer-aided diagnosis is reportedly a potential measurement tool for screening lung nodules, with quality interpretation and fewer variabilities among readers.^10,11,12,13^ The European Society of Radiology and European Respiratory Society recommend computer-aided diagnosis of lung cancer nodules.^14^ The investigated computed tomography scans were considered low dose at 3 mSv or less; however, the requirement for low-dose computed tomography is actually < 2 mSv.^5,15^ However, computer-aided diagnosis in computed tomography can miss lung cancer nodules that are detected by radiologists.^11^ A computer-aided diagnosis system has less sensitivity for ground-glass nodules than the conventional reading system.^16^ Computer-aided diagnosis systems often miss lesions that are large, endobronchial, and inseparable from the mediastinum or perihilar. In addition, computer-aided diagnosis is typically used to aid radiologists in screening trials; therefore, both methods are used in clinical practice. Hence, the feasibility and efficacy of computer-aided diagnosis in computed tomography for lung cancer nodules should be investigated in detail.

## OBJECTIVE

In this retrospective study, we aimed to compare the diagnostic parameters of low-dose (< 2 mSv) computed tomography interpretation results using a computer-aided diagnosis system for lung cancer screening with those of a conventional reading system by radiologists.

## METHODS

### Ethics approval and consent to participate

The present study involved chart reviews from a database (of lung cancer diagnosis) of chest computed tomography images of patients at risk for lung cancer. Therefore, the requirements for ethics approval from The First Affiliated Hospital of Nanchang Medical College Review Board, consent to participate, consent to publish, and registration in the Chinese Clinical Trial Registry were waived by Xianyang Cai-Hong Hospital (China), Hospital of Shaanxi University of Chinese Medicine (China), and Jiangxi Provincial People’s Hospital Affiliated with The First Affiliated Hospital of Nanchang Medical College (China).

### Study population

**Figure 1:**
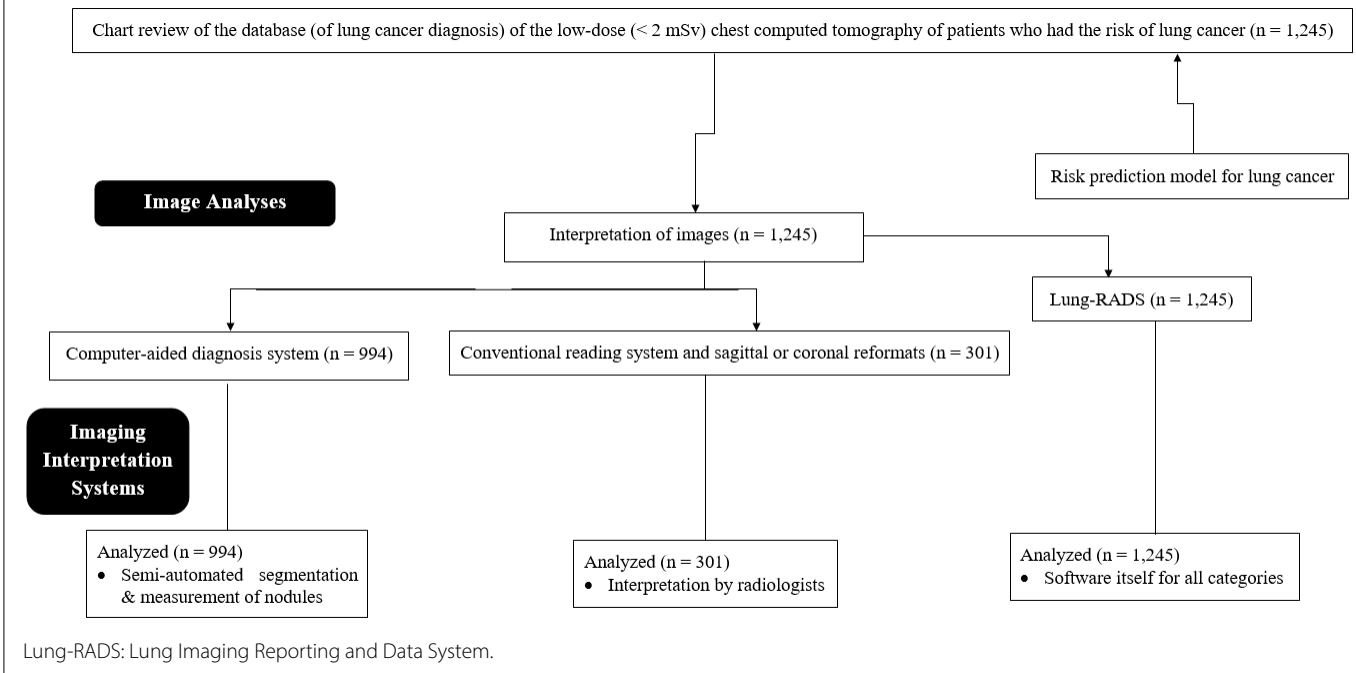
Flowchart of patient selection.

Low-dose (< 2 mSv) chest computed tomography images of patients at risk of lung cancer according to the risk prediction model, including demographics and metabolic markers for lung cancer,^17^ from the radiology departments of Xianyang Cai-Hong Hospital, Hospital of Shaanxi University of Chinese Medicine, and The First Affiliated Hospital of Nanchang Medical College from December 8, 2019, to January 1, 2021 were included in the analyses. Patients without nodules were excluded from this study. A flowchart of the patient selection is shown in Figure 1.

Lung-RADS: Lung Imaging Reporting and Data System.

### Imaging protocols of chest computed tomography

The detailed protocols for chest computed tomography were based on individual institutional guidelines; there were no differences between the image acquisition protocols and basic characteristics of each center. The basic configuration comprised a computed tomography scanner with at least 16 detector rows. A whole thoracic scan was performed with a one-breath hold at full inspiration. The slice thickness was 1.5 mm, and the image acquisition settings were 80–120 kVp, 22 mA, and the lowest possible collimation on the scanner; the radiation dose was less than 2 mSv.

### Computed tomography image analyses

#### Computer-aided diagnosis system

The AVIEW LCS Lung Cancer Screening SW system (Coreline Europe GmbH, Eschborn, Germany) was available at the three institutions. All chest computed tomography scans were uploaded to the cloud included with the software. All participating radiologists interpreted the chest computed tomography scans irrespective of the availability of the software. Interpretations of the chest computed tomography scans were based on a computer-aided diagnosis system for lung nodules (Visia^™^, MeVis Medical Solutions AG, Bremen, Germany), including semi-automated segmentation and measurement of the nodules (the diameter of the nodules was automatically measured by automatic segmentation).

#### Conventional reading system

The computed tomography images were initially screened for interpretations using the institutional conventional system, and other reformats (sagittal or coronal) were accessible to the radiologists, who had a minimum of three years of experience in thoracic imaging, at each hospital. The nodule diameters were measured manually using an electronic caliper (DIGITAL CALIPER, Model No. DT-300/D-300W, Niigata seiki Co., Ltd., Sanjo, Niigata, Japan).

### Lung Imaging Reporting and Data System

The chest computed tomography scan interpretations were based on the Lung Imaging Reporting and Data System (Lung-RADS) Version 1.1.^18^ The software displays the Lung-RADS category results. The predictions of the different Lung-RADS categories are presented in Table 1.

**Table 1.** Lung Imaging Reporting and Data System category distribution

Parameters	Predicted categories
No computed tomography images available	0 (Incomplete)
No appearances of nodules in computed tomography images	1 (Absent)
< 6 mm ɸ for solid nodules and part-solid nodules	2 (Benign)
≥ 6 to < 8 mm ɸ for solid nodules and ≥ 6 ɸ with solid component < 6 mm for part-solid nodules	3 (Probably benign)
≥ 8 to < 15 mm ɸ for solid nodules and ≥ 6 ɸ with solid components ≥ 6 mm to < 8 mm for part-solid nodules	4A (Suspicious)
≥ 15 mm ɸ for solid nodules and > 8 ɸ with solid components ≥ 8 mm for part-solid nodules	4B (Very suspicious)
Suspicious nodules with additional features in imaging analysis	4X (Very suspicious)
Significant clinical and imaging parameters	4S (Clinically significant)

ɸ: diameter (mean diameter of both the long and short axis) according to the 2019 American College of Radiology guidelines.

### Statistical analysis

InStat 3.01 (GraphPad Software, San Diego, California, United States) was used for statistical analysis. Continuous data were compared using the Mann-Whitney *U* test, unpaired *t*-test with Kolmogorov-Smirnov test, or one-way analysis of variance. Categorical data were compared using the chi-square test for independence (for comparisons of more than two classes) or Fisher’s exact test (for comparisons of two classes).^5^ Tukey-Kramer multiple comparisons tests (considering a critical value [*q*] > 3.314 as significant) were performed for *post hoc* analysis. McNemar’s tests were used to compare diagnostic parameters between the two systems.^5^ P values less than 0.05 were considered statistically significant.

## RESULTS

### Characteristics of participants and nodules

A database of 1,245 patients was retrospectively reviewed. Among them, a database of 301 patients was viewed using the conventional reading system with the radiologists unaware of the computer-aided diagnosis system data. In addition, the data of 944 patients were viewed using a computer-aided diagnosis system without any subsequent review by a radiologist. Details of the participants’ characteristics are presented in Table 2. A total of 226 nodules among the database of 301 patients were detected by radiologists using the conventional reading system, and 1,048 nodules in the database among 944 patients were detected using the computer-aided diagnosis system. The numbers of detected nodules per patient (P < 0.0001, Fisher’s test) and solid nodules (P < 0.0001, Fisher’s test) were higher in the database of patients evaluated with the computer-aided diagnosis systems compared with those with the conventional reading system. The number of pure-ground nodules was fewer in the database of patients evaluated with the computer-aided diagnosis system compared with patients evaluated with the conventional reading system (P = 0.0003, Fisher’s test). The nodule size in the transverse plane detected by the conventional reading and computer-aided diagnosis systems was 4.41 ± 1.22 mm and 4.32 ± 1.85 mm, respectively, and 4.61 ± 2.05 mm and 4.92 ± 1.81 mm in the maximum orthogonal plane, respectively. The size of the nodules was reported as the maximum in any plane for the computer-aided diagnosis system. The nodule characteristics are presented in Table 3.

**Table 2.** Participants characteristics

Characteristics		Conventional reading system by radiologists	Computer-aided diagnosis system	Comparisons			
Numbers of patients included in the analysis		301	944	P value	95% Cl	Df	F value
**Sex**	Male	281 (93)	873 (92)	0.7032 (Fisher’s test)	0.7424–1.6529	N/A	N/A
	Female	20 (7)	71 (8)				
**Age (years)**		61.15 ± 8.14	62.15 ± 8.55	0.0741 (*t*-test)	N/A	1243	1.1030
**Smoking status**	Current	15 (5)	55 (6)	0.8054 (χ^2^-test)	N/A	2	N/A
	Previous	126 (42)	381 (40)				
	None	160 (53)	508 (54)				
**Participants with available prior computed tomography**		51 (17)	141 (15)	0.4099 (Fisher’s test)	0.8636–1.4499	N/A	N/A

Continuous data are presented as the mean ± standard deviation and constant data are presented as the frequency (percentage). Continuous data were compared using a *t*-test, and categorical data were compared using the chi-square test for independence or Fisher’s exact test for statistical analysis. Results were considered significant if the P value was less than 0.05. Cl = confidence interval; Df = degree of freedom; N/A = not applicable; χ^2^-test = chi-square test.

**Table 3.** Nodule characteristics

Characteristics		Conventional reading system by radiologists	Computer-aided diagnosis system	Comparisons		
Total numbers of nodules included in the analysis		226	1,048	P value	95% Cl	F value
**Nodule(s)/Patient**		0.75	1.11*	< 0.0001 (Fisher’s test)	0.6108 – 0.8256	N/A
**Characters of nodules**						
**Solid**		180 (80)	943 (90)*	< 0.0001 (Fisher’s test)	0.3994 – 0.6932	N/A
**Part-solid**		18 (8)	50 (5)	0.0707 (Fisher’s test)	1.0130 – 2.3240	N/A
**Pure-ground glass**		2 8 (12)*	55 (5)	0.0003 (Fisher’s test)	1.4630 – 2.8150	N/A
**Size (mm)**						
**Transverse plane**		4.41 ± 1.22	4.32 ± 1.85	0.4846 (*t*-test)	-0.3425 – 0.1625	2.2990
**Maximum orthogonal plane**		N/A	4.61 ± 2.05	N/A	N/A	N/A
**Any maximum plane**		N/A	4.92 ± 1.81*	N/A	N/A	N/A
**Comparison for size**						
**P value**		N/A	< 0.0001 (ANOVA; F value: 25.9670)	N/A	N/A	N/A
**q value**	Transverse plane versus maximum orthogonal plane	N/A	4.9250 (95% Cl: -0.4851 – -0.0949)	N/A	N/A	N/A
	Transverse plane versus any maximum plane	N/A	10.1900 (95% Cl: -0.7951 – -0.4049)	N/A	N/A	N/A
	Maximum orthogonal plane versus any maximum plane	N/A	5.2650 (95% Cl: -0.5051 – -0.1149)	N/A	N/A	N/A

Continuous data are presented as the mean ± standard deviation and categorical data are presented as the frequency (percentage). Continuous data were compared using an unpaired *t*-test or one-way analysis of variance and categorical data were compared using Fisher’s exact test for statistical analysis. Tukey-Kramer multiple comparisons test was used for *post hoc* analysis. Results were considered significant if the P value was less than 0.05 and *q*-value was greater than 3.314. *Significant difference. ANOVA = analysis of variance; Cl = confidence interval; N/A = not applicable. Size = to calculate the nodule mean diameter, we measured both the long and short axes to two decimal points and reported the mean nodule diameter to two decimal points.

### Lung-RADS category distribution and positivity rates

A total of 20 (7%) and 102 (11%) patients were diagnosed with cancer using the conventional reading and computer-aided diagnosis systems, respectively. The computer-aided diagnosis system detected a higher number of patients with cancer than the conventional reading system (P = 0.0345, Fisher’s test). If nodules were measured in a transverse plane, there were no significant differences between the two systems in the number of patients diagnosed with cancer (P = 0.6150, Fisher’s test). However, if nodules were measured in any maximum plane with the computer-aided diagnosis system, a higher number of patients with cancers were detected than with the transverse plane measurement using the conventional reading or computer-aided diagnosis systems. The details of the per-patient Lung-RADS category distribution screening results for lung cancers are presented in Table 4.

**Table 4.** Per patient Lung-RADS category distribution, screening results, and lung cancers

Lung-RADS category		Conventional reading system by radiologists	Computer-aided diagnosis system		
		Transverse plane	Transverse plane	Maximum orthogonal plane	Any maximum plane
**Number of patients included in analysis**		301	944	944	944
**1**	Numbers of patients	181 (60)	47 8 (51)	461 (49)	472 (50)
	Number of patients with cancer	0 (0)	0 (0)	0 (0)	0 (0)
**2**	Number of patients	91 (30)	352 (37)	363 (38)	368 (39)
	Number of patients with cancer	7 (2)	18 (2)	25 (3)	42 (4)
**3**	Number of patients	17 (6)	55 (6)	69 (7)	56 (6)
	Number of patients with cancer	4 (1)	12 (1)	14 (1)	19 (2)
**4A**	Number of patients	7 (2)	31 (3)	35 (4)	31 (3)
	Number of patients with cancer	5 (2)	21 (2)	23 (2)	28 (3)
**4B**	Number of patients	3 (1)	11 (1)	12 (1)	13 (1)
	Number of patients with cancer	2 (1)	8 (1)	9 (1)	10 (1)
**4X**	Number of patients	2 (1)	17 (2)	4 (1)	4 (1)
	Number of patients with cancer	2 (1)	11 (1)	2 (1)	3 (1)
**Total number of patients with cancers**		20 (7)	70 (7)	73 (8)	102 (11)
**Comparison of total number of patients with cancer**		P value			95% Cl
**Transverse plane in the conventional reading system versus transverse plane in the computer-aided diagnosis system**		0.7032			0.6124–1.3620
**Transverse plane in the conventional reading system versus maximum orthogonal plane in the computer-aided diagnosis system**		0.6150			0.5901–1.3170
**Transverse plane in the conventional reading system versus any maximum plane in the computer-aided diagnosis system**		0.0345			0.4333–0.9906
**Transverse plane in the computer-aided diagnosis system versus maximum orthogonal plane in the computer-aided diagnosis system**		0.8620			0.8214–1.1630
**Transverse plane in the computer-aided diagnosis system versus any maximum plane in the computer-aided diagnosis system**		0.0130			0.6632–0.9627
**Maximum orthogonal plane in the computer-aided diagnosis system versus any maximum plane in the computer-aided diagnosis system**		0.0260			0.6844–0.9834

Data are presented as the frequency (percentage). Fisher’s exact test was used for statistical analysis. Results were considered significant if the P value was less than 0.05. Cl = confidence interval; Lung-RADS = Lung Imaging Reporting and Data System; N/A = not applicable. Bold values represent statistical significance.

A total of 17 (8%) and 154 (15%) nodules were diagnosed using the conventional reading and computer-aided diagnosis systems, respectively (P = 0.0035, Fisher’s test). If nodules were measured in a transverse plane, there were no significant differences between the two systems in the number of nodules diagnosed with cancer (P = 0.6921, Fisher’s test). However, if nodules were measured in any maximum plane with the computer-aided diagnosis system, then higher numbers of cancerous nodules were detected compared with the transverse plane measurement using the conventional reading or computer-aided diagnosis systems. The details of the per-nodule Lung-RADS category distribution screening results and lung cancers are presented in Table 5.

**Table 5.** Per nodule Lung-RADS category distribution, screening results, and lung cancers

Lung-RADS category		Conventional reading system by radiologists	Computer-aided diagnosis system		
		Transverse plane	Transverse plane	Maximum orthogonal plane	Any maximum plane
**Number of nodules included in analysis**		226	1,048	1,048	1,048
**2**	Number of nodules	189 (84)	905 (86)	864 (82)	785 (75)
	Number of nodules with cancer	5 (2)	65 (6)	57 (5)	65 (6)
**3**	Number of nodules	20 (9)	71 (7)	105 (10)	161 (15)
	Number of nodules with cancer	1 (1)	12 (1)	24 (2)	31 (3)
**4A**	Number of nodules	8 (4)	45 (4)	32 (3)	52 (5)
	Number of nodules with cancer	5 (2)	9 (1)	22 (2)	29 (3)
**4B**	Number of nodules	7 (3)	18 (2)	36 (3)	35 (3)
	Number of nodules with cancer	5 (2)	2 (0.5)	18 (2)	22 (2)
**4X**	Number of nodules	2 (1)	9 (1)	11 (1)	15 (1)
	Number of nodules with cancer	1 (1)	2 (0.5)	5 (1)	7 (1)
**Total number of nodules with cancer**		17 (8)	90 (9)	126 (12)	154 (15)
**Comparison of total number of nodules with cancer**		P value		95% Cl	
**Transverse plane in the conventional reading system versus transverse plane in the computer-aided diagnosis system**		0.6921		0.5639–1.396	
**Transverse plane in the conventional reading system versus maximum orthogonal plane in the computer-aided diagnosis system**		0.0622		0.4050–1.022	
**Transverse plane in the conventional reading system versus any maximum plane in the computer-aided diagnosis system**		0.0035		0.3288–0.8373	
**Transverse plane in the computer-aided diagnosis system versus maximum orthogonal plane in the computer-aided diagnosis system**		0.0119		0.6940–0.9633	
**Transverse plane in the computer-aided diagnosis system versus any maximum plane in the computer-aided diagnosis system**		< 0.0001		0.6016–0.8452	
**Maximum orthogonal plane in the computer-aided diagnosis system versus any maximum plane in the computer-aided diagnosis system**		0.0830		0.7727–1.0170	

Data are demonstrated as the frequency (percentage). Fisher’s exact test was used for statistical analysis. Results were considered significant if the P value was less than 0.05. Cl = confidence interval; Lung-RADS = Lung Imaging Reporting and Data System; N/A = not applicable. Bold values represent statistical significance.

### Diagnostic parameters

Sensitivity and positive predictive values were higher if nodules were measured in any maximum plane of the computer-aided diagnosis system compared with that measured in any plane of any system. The sensitivity, specificity, and positive predictive values did not differ between the transverse plane in the conventional reading and computer-aided diagnosis systems, transverse plane in the conventional reading system, and maximum orthogonal plane in the computer-aided diagnosis system. The details of the diagnostic parameters for the imaging interpretation systems for lung cancer are presented in Table 6.

**Table 6.** Diagnostic parameters for imaging interpretation systems for lung cancer

Parameters	CRS by radiologists	CAD			Comparisons					
					P value					
	TP (%)	TP	MOP	AMP	TP of CRS vs. TP of CAD	TP of CRS vs. MOP of CAD	TP of CRS vs. AMP of CAD	TP of CAD vs. MOP of CAD	TP of CAD vs. AMP of CAD	MOP of CAD vs. AMP of CAD
Sensitivity	92.69% (68.21–99.81%)	92.61% (67.89–99.11%)	95.87% (70.12–99.15%)	96.15% (71.12–99.85%)	0.8541	0.0612	0.0431	0.0581	0.0411	0.0981
Specificity	89.91% (88.12–92.15%)	88.11% (86.15–91.11%)	87.12% (84.11–89.99%)	82.98% (80.11–88.15%)	0.9121	0.0852	0.0421	0.0651	0.0391	0.0382
PPV	7.52% (5.15–9.15%)	8.59% (7.11–10.12%)	12.02% (10.15–14.11%)	14.69% (11.12–16.52%)	0.6891	0.0611	0.0041	0.0131	< 0.0001	0.0891
NPV	99.81% (98.12–100%)	99.82% (98.08–99.89%)	99.81% (98.15–99.81%)	99.89% (98.86–99.89%)	0.5831	0.6211	0.5541	0.6541	0.6321	0.6641

Parameters are presented as the mean (range). McNemar’s tests were used to compare parameters. Results were considered significant if the P value was less than 0.05. CRS = conventional reading system; CAD = computer-aided diagnosis system; TP = transverse plane; MOP = maximum orthogonal plane; AMP = maximum plane; PPV = positive predictive value; NPV = negative predictive value.

## DISCUSSION

This study revealed that the sensitivity and positive predictive values were higher if nodules were measured with the computer-aided diagnosis system than those measured with the conventional reading system. The diagnostic parameter results of the current study are consistent with those of previous retrospective studies.^5,15^ Small nodules missed using a conventional reading system can be detected by the computer-aided diagnosis system.^15,19^ The computer-aided diagnosis system facilitates the diagnosis of cancerous nodules in patients at risk of lung cancer.

We found that the specificity and negative predictive values were the same when nodules were measured with the computer-aided diagnosis or conventional reading systems. There was a difference in nodule sizes measured by the two systems. The radiologists did not measure oversized nodules, and the computer-aided diagnosis system did not measure undersized nodules. Nodule size and the risk of lung cancer are separate issues that require investigation.

In this study, we found that the Lung-RADS screening rate per patient was higher with the computer-aided diagnosis system than that of the conventional reading system. In addition, the Lung-RADS screening rates per nodule differed between imaging interpretation systems for lung cancer. The per patient and per nodule Lung-RADS screening rates in the current study were inconsistent with those of retrospective studies.^5,15^ The increased diagnosis of small nodules with cancer resulted in a higher per patient Lung-RADS screening rate. Moreover, the increased diagnosis rate of small nodules significantly changed the per-nodule Lung-RADS screening rate. The use of data from more than one institution, heterogeneity of the patients,^20^ and higher numbers of involved radiologists^21^ may explain the contradictory results between imaging interpretation systems for lung cancer in the current study and those of other retrospective studies.^5,15^ A computer-aided diagnosis system is a more accurate tool for lung cancer screening among at-risk patients.

We found that the diagnostic parameters did not differ between the transverse plane in the conventional reading and computer-aided diagnosis systems, transverse plane of the conventional reading system, and maximum orthogonal plane of the computer-aided diagnosis system. The results of the different planes using the computer-aided diagnosis system in the current study were consistent with those of a previous retrospective study.^5,22^ Lung cancer can be missed by radiologists using computer-aided diagnosis systems.^11^ Lung-RADS does not recommend any specific plane in computed tomography imaging for the measurement of nodules,^5^ although Lung-RADS Version 1.1^18^ is validated in the transverse plane. However, nodules measured in the transverse plane cannot reflect the actual nodule size.^23^ Low-dose noncontrast computed tomography images are also responsible for insignificant results.^19^ Further research is required to overcome missed lung cancer nodules, and clear instructions are required for the specific planes in computed tomography imaging for the measurement of nodules in lung cancer screening.

The current study revealed significantly fewer pure ground nodules and significantly more solid nodules among patients evaluated by the computer-aided diagnosis system compared with patients evaluated by the conventional reading system. The pure-ground and solid nodule results observed in the current study were consistent with those of other retrospective studies.^5,15^ A computer-aided diagnosis system has less sensitivity for ground-glass nodules than that of the conventional reading system.^16^ Solid nodules that can be detected by a computer-aided diagnosis system are sometimes missed by radiologists.^15,19^ The conventional reading system is recommended for pure-ground nodules, whereas computer-aided diagnosis systems are recommended for solid nodules in lung cancer screening among at-risk patients.

We also found insignificant differences in part-solid nodules between patients evaluated with the computer-aided diagnosis and conventional reading systems. The performance results of the imaging systems for the detection of part-solid nodules in the current study were consistent with those of a prospective multicenter study.^24^ The conventional reading system showed comparable performance to the computer-aided diagnosis system for part-solid nodules.

This was an interesting study on a highly relevant topic that included a large database with follow-up data on malignancy diagnoses. This study had some limitations, mainly its retrospective design (the datasets of the conventional reading and computer-aided diagnosis systems were different) and lack of cross-sectional analysis. It may be more valuable to compare the performance of both systems using the same dataset. However, the gold standard (biopsy, surgical pathology, or position emission tomography) has not yet been described. This study noted that the data were organized according to a local “risk prediction model” established for a single institution. This is problematic as it did not translate to other universally standardized classifications (United States Preventive Services Task criteria). There was an apparent difference between the small number of cases (n = 301) read by radiologists and an entirely different large (n = 944) set of cases read by the computer. Consequently, this study included the two separate, albeit overlapping, issues of diagnosis and measurement. This might be responsible for the overall differences between the radiologists’ and computer’s results. A possible justification for this is that the study included clinical features, which showed broad similarities between the patients diagnosed by radiologists and patients diagnosed by computer (P > 0.05). This study used size in maximum length rather than volume, which is not conventionally used when screening populations in the United Kingdom. The possible justification for this is that nodule diameter or volume can be used for lung cancer screening.^25^ When comparing radiologist interpretations and computer-aided diagnoses it is critical to use the same images. Given that there were two different image sets in this study, it was not possible to validate their performance because there were many different variables between the two groups. Therefore, the increased diagnosis of lung cancer using a computer-aided system may also reflect differences in underlying risks among patients.

## CONCLUSIONS

This study validates a commercial computer-aided diagnosis system (Lung-RADS) in a clinical setting, tackling an important question on the utility of computer-aided diagnosis of nodules in the evaluation of computed tomography scans. Use of a computer-aided diagnosis system in low-dose computed tomography (< 2 mSv) for lung cancer screening resulted in higher per-patient and per-nodule Lung-RADS screening rates among patients at risk of lung cancer. Therefore, we recommend a computer-aided diagnosis system for lung cancer screening with low-dose (< 2 mSv) computed tomography, especially for solid nodules. In addition, clear instructions are required regarding the specific plane measured in computed tomography imaging for lung cancer nodule screening. Further investigation of diagnosis rates and measurement accuracy in ultra-low-dose computed tomography (< 1 mSv and < 0.5 mSv) may be of interest.
